# The Impact of the Duration of Type 2 Diabetes Mellitus on Biochemical and Oxidative Stress Parameters and Renal Function

**DOI:** 10.7759/cureus.87619

**Published:** 2025-07-09

**Authors:** Sabina Zukic, Sadeta Hadzic, Nermina Babic, Nesina Avdagic, Sabaheta Hasic, Salih Azabagic

**Affiliations:** 1 Department of Family Medicine, Banovici Health Center, Banovici, BIH; 2 Department for Hygienic and Epidemiological Surveillance, University Clinical Center Tuzla, Tuzla, BIH; 3 Department of Human Physiology, University of Sarajevo, Faculty of Medicine, Sarajevo, BIH; 4 Department of Medical Biochemistry, University of Sarajevo, Faculty of Medicine, Sarajevo, BIH; 5 Department of Internal Medicine, University Clinical Center Tuzla, Tuzla, BIH

**Keywords:** diabetes mellitus, glomerular filtration, oxidative stress, total antioxidant capacity, von willebrand factor

## Abstract

Background and objective

Type 2 diabetes mellitus (T2DM) is a global health issue that has seen a significant increase in prevalence worldwide. Oxidative stress plays a crucial role in the pathogenesis of numerous chronic diseases. Oxidative stress induced by hyperglycemia has a central role in the development of insulin resistance, as well as micro- and macrovascular complications of diabetes mellitus. This study aimed to investigate the influence of the duration of T2DM on blood glucose levels, glycated hemoglobin (HbA1c), renal function parameters, oxidative stress, and von Willebrand factor (vWf) activity in individuals with diabetes.

Methodology

A total of 135 participants from both genders with T2DM were included in this study. The participants were divided into three groups based on the duration of their disease: up to five years (46 participants), from 6-10 years (49 participants), and over 10 years (40 participants). The investigated parameters were as follows: fasting glucose, two-hour postprandial glucose, HbA1c, total antioxidant capacity (TAC), glomerular filtration rate (GFR), and vWf activity. Statistical analysis was performed using SPSS Statistics (IBM Corp., Armonk, NY). The Kolmogorov-Smirnov test was applied to assess the normality of distribution. Differences between the groups were analyzed using the Kruskal-Wallis test and analysis of variance (ANOVA), with appropriate post-hoc tests. A p-value <0.05 was considered statistically significant.

Results

The average age of the participants was 60.86 ± 8.87 years, the average weight was 86 ± 14.6 kg, the average height was 168 ± 9.18 cm, the waist circumference was 99 ± 11.4 cm, the systolic blood pressure was 127 ± 15.6 mmHg, and the diastolic blood pressure was 77 ± 6.7 mmHg. The study revealed statistically significant differences between the groups (p<0.05) for the following parameters: two-hour postprandial glucose (p=0.001), HbA1c (p=0.048), GFR (p=0.038), and vWf activity (p=0.006). No statistical significance was found for TAC values (p>0.05).

Conclusions

Higher levels of vWf activity were found in people who had been treated for type 2 diabetes for more than 10 years. These findings indicate that the level of vWf activity in people with type 2 diabetes 10 years after the onset of the disease can be used as a marker of vascular pathology.

## Introduction

Type 2 diabetes mellitus (T2DM) is a global health issue, and there has been a significant increase in its prevalence worldwide. The most important complications of diabetes are diabetic nephropathy, cardiovascular diseases, and neurological complications, all of which affect the quality of life in people with diabetes, the outcome of the disease, and healthcare costs [1,2]. Oxidative stress plays a key role in the pathogenesis of numerous chronic diseases. Free radicals serve important physiological functions in molecular processes, such as cell communication, synaptic plasticity, defense against pathogen invasion, memory formation, apoptosis, cell proliferation, aging, and autophagy. Oxidative stress occurs when the levels of free radicals exceed the natural antioxidant defense capacity (TAC) in the body and thus cause tissue dysfunction. TAC determines the total antioxidant capacity. Oxidative stress is a state of imbalance between oxidants and antioxidants. Oxidative stress induced by hyperglycemia has a central role in the development of insulin resistance, as well as microvascular and macrovascular complications of diabetes mellitus. Modern lifestyles associated with processed foods, exposure to a wide range of chemicals, and lack of physical activity play a significant role in inducing oxidative stress [3,4].

One of the consequences of oxidative stress, which has an influence on gene expression in general and cell survival, is DNA damage. In addition to its direct harmful effects, free radicals can damage cells indirectly by activating several stress-sensitive intracellular signaling pathways. In people with diabetes, stress plays a key role by altering lipid peroxidation and inducing mitochondrial dysfunction and DNA damage. Furthermore, oxidative stress has various roles in pathological situations, as well as in age-related diseases such as cancer, chronic kidney disease, chronic obstructive pulmonary disease, and cardiovascular diseases. The continuous loss of tissue function due to various causes, including an increase in the number of free radicals, is referred to as aging and the diseases associated with it. The theory of oxidative stress is widely accepted as a primary explanation for the aging process and the difficulties associated with it. Therefore, maintaining a normal redox state in biology is crucial for preventing problems caused by oxidative stress, as well as insulin resistance [5].

Von Willebrand factor (vWf) plays a significant role in primary hemostasis, participating in platelet adhesion and aggregation at the site of vascular injury, and secondary hemostasis, as a protein carrier for factor VIII of the blood coagulation cascade. vWf is involved in the pathogenesis of cardiovascular diseases, contributing to the development of microvascular and macrovascular complications in diabetes [6]. Horvath et al. [7] highlighted that vWF activity may serve as an indicator of vascular disease progression. In examining various biological markers, Constans and Conri [8] established that vWf is the best endothelial biomarker and a predictor of ischemic heart disease and stroke risk. Vascular complications are the leading causes of death and disability in people with T2DM. Diabetes poses a significant burden on healthcare systems due to its high prevalence and the associated treatment costs. Early detection of diabetes and cardiovascular diseases is crucial for reducing morbidity, mortality, and the societal and economic impact of diabetes-related burdens on the population. Data show that primary and secondary prevention are key to reducing cardiovascular incidents and improving life expectancy in people with diabetes, and that better glucose control reduces disease complications [9,10].

This study aimed to examine the impact of the duration of T2DM on blood glucose levels, glycated hemoglobin (HbA1c), kidney function parameters, oxidative stress, and vWf activity.

## Materials and methods

Study design

This study employed a cross-sectional design, analyzing retrospective data during the period from 2017 to 2022.

Subjects

Subjects with a previously confirmed diagnosis of T2DM who used therapy, oral, insulin, and combined oral and insulin therapy were included in the study. The criteria for inclusion in the study were as follows: subjects with a confirmed diagnosis of T2DM who voluntarily agreed to participate in the study and provided written consent. The exclusion criteria were as follows: subjects who refused to participate in the study; subjects with incomplete laboratory data; subjects with malignant diseases; those with macroalbuminuria, rheumatoid arthritis, anemia, thrombocytopenia, liver diseases, and hemophilia; subjects on anticoagulant therapy; those with obstructive lung diseases; subjects with inflammatory diseases; subjects with extreme changes in body or muscle mass; subjects with amputated limbs; and pregnant women. This study ultimately included participants with T2DM of both sexes. A total of 135 participants were included, who were divided into three groups based on the duration of their diabetes: up to five years (46 participants), from 6 to 10 years (49 participants), and over 10 years (40 participants). All participants signed an informed written consent, and the study was conducted in accordance with the principles of the Declaration of Helsinki. The study was approved by the Ethics Committee of Health Centers Banovici and Zivinice.

Demographic and clinical parameters

The following data were collected from all subjects who met the criteria for inclusion in the study: age, sex, anthropometric parameters, duration of the disease, fasting glucose values (glucose reference values up to 7.0 mmol/l), glucose values two hours after eating (reference values up to 11.1 mmol/l), HbA1c (reference values up to 6.5%), urea (reference values for women from 3.5-7.2 mmol/L and for man from 3.0-9.2 mmol/L), creatinine (reference values for women from 44-80 mmol/L and for men 64-104mmol/L), vWf activity (reference values from 0.50-1.50 IU); blood pressure was measured (reference values up to 130/80 mmHg), and a blood sample was taken from the cubital vein.

Collecting blood samples and lab tests

Blood samples of all subjects were taken by puncture of the cubital vein on an empty stomach. Biochemica analyses and other tests were performed. All subjects underwent routine laboratory tests of hematological and biochemical parameters: complete blood count, urea, creatinine, fasting and two-hour postprandial glucose concentration, and HbA1c in the blood, and urine microalbuminuria. All of the above tests were performed as part of the usual laboratory workup of subjects with diabetes mellitus, using standard methods on automatic analyzers, in the laboratory of the Banovići and Zivinice Health Centers. Routine analyses were performed immediately, and the remaining serum was stored in a freezer at -25 °C until determination of the concentration of TAC. TAC in serum was determined spectrophotometrically using an appropriate commercial test, according to the manufacturer's instructions. Blood samples for the determination of vWf activity were taken in a tube with the anticoagulant Na citrate. After centrifugation, plasma was separated, and vWf activity was determined at the Institute for Transfusion Medicine of the Federation of Bosnia and Herzegovina.

Estimation of the Level of Glomerular Filtration

Estimated glomerular filtration rate (eGFR) was determined using a predictive equation developed from the MDRD (Modification of Diet in Renal Disease) study (R). For this purpose, the following data were used: serum creatinine concentration value, patient's age, gender, and race. The MDRD equation is standardized to an average body surface area of 1.73 m^2^: 

eGFR (ml/min/1.73 m2) = 175 × [Scr (μmol/l) × 0.011312]-1.154 × [age]-0.203 × [1.212 for African Americans] × [0.742 for females]

[Scr: serum creatinine concentration (μmol/l), age: age of the subject in years]

Statistical analysis

Statistical analysis was performed using SPSS Statistics (IBM Corp., Armonk, NY). The Kolmogorov-Smirnov test was applied to assess the normality of distribution. Differences between the groups were determined using the Kruskal-Wallis test or analysis of variance (ANOVA), along with appropriate post-hoc tests. A p-value <0.05 was considered statistically significant.

## Results

**Figure 1:**
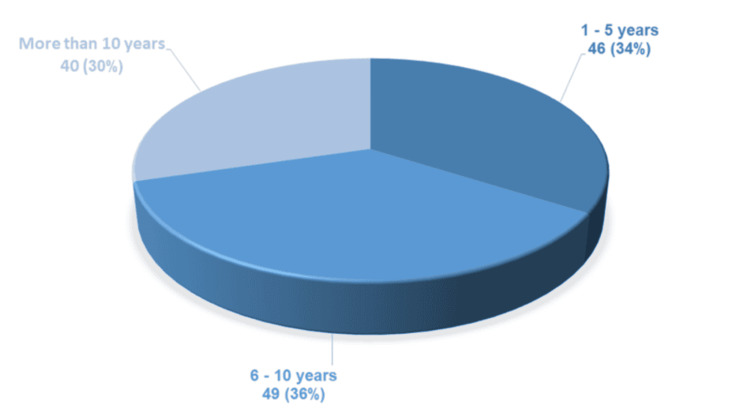
Distribution of participants according to the duration of illness

Data from 135 participants were analyzed, who were divided into three groups based on the duration of diabetes mellitus (Figure 1). The average age of the participants was 60.86 ± 8.87 years; the average weight was 86 ± 14.6 kg; the average height was 168 ± 9.18 cm; the systolic blood pressure was 127 ± 15.6 mmHg; and the diastolic blood pressure was 77 ± 6.7 mmHg.

As shown in Table 1, the lowest mean value of the HbA1c parameter was recorded in participants with a disease duration of one to five years, whereas the highest mean value was observed in the third group of participants (disease duration longer than 10 years). For fasting glucose and glucose measured two hours postprandially, the highest mean values were recorded in the second group (disease duration of 6-10 years), while the lowest mean values of these indicators were observed in the first group of participants with a disease duration of one to five years.

**Table 1:** Descriptive statistical parameters for analyzed variables GFR: glomerular filtration rate; HbA1c: glycated hemoglobin; TAC: total antioxidant capacity; SD: standard deviation

Analyzed variables	Participant group	Number of participants	Mean ± SD	Min	Max	Variation range	Kruskal-Wallis test	df	P-value
HbA1c	1–5 years	46	6.71 ± 1.37	2.00	10.00	8.00	6.09	2	0.048
	6–10 years	49	7.50 ± 1.52	2.00	11.00	9.00			
	More than 10 years	40	7.74 ± 1.46	6.00	12.00	6.00			
	Total	135	6.89 ± 1.53	2.00	12.00	10.00			
Fasting glucose	1–5 years	46	8.17 ± 1.89	5.00	15.00	10.00	5.72	2	0.057
	6–10 years	49	9.75 ± 3.28	4.00	18.00	14.00			
	More than 10 years	40	9.21 ± 3.71	4.00	26.00	22.00			
	Total	135	8.18 ± 3.11	4.00	26.00	22.00			
Postprandial glucose	1–5 years	46	10.90 ± 3.70	4.00	21.00	17.00	15.16	2	0.001
	6–10 years	49	13.40 ± 5.76	4.00	35.00	31.00			
	More than 10 years	40	12.10 ± 5.34	6.00	31.00	25.00			
	Total	135	10.65 ± 5.25	4.00	35.00	31.00			
TAC	1–5 years	46	276.12 ± 54.56	110.35	405.74	295.39	1.54	2	0.464
	6–10 years	49	294.36 ± 66.86	82.40	438.24	355.84			
	More than 10 years	40	280.74 ± 66.56	37.40	392.62	355.22			
	Total	135	287.96 ± 65.82	37.40	447.90	410.50			
GFR	1–5 years	46	93.50 ± 19.07	60.00	138.00	78.00	6.52	2	0.038
	6–10 years	49	94.44 ± 33.96	40.00	281.00	241.00			
	More than 10 years	40	80.97 ± 22.08	28.00	116.00	88.00			
	Total	135	91.30 ± 0.00	28.00	281.00	253.00			

**Figure 2:**
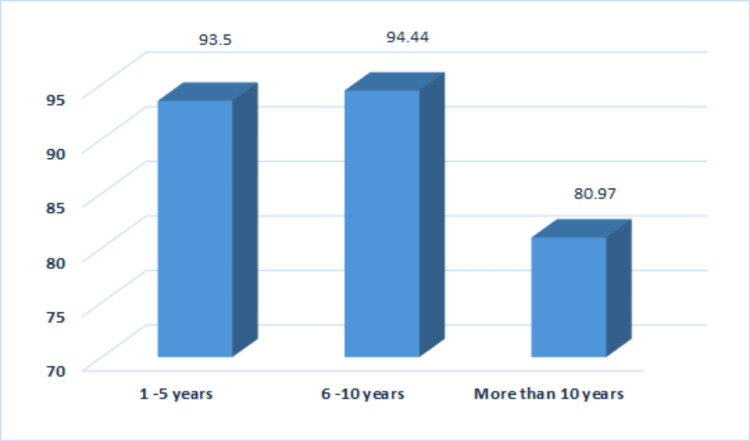
Glomerular filtration rate according to disease duration

The highest mean value for TAC was observed in the second group, which was also the case for glomerular filtration rate (GFR) (Table 1). The lowest mean value of TAC was seen in the first group of participants (Table 1), while the lowest mean value of GFR was recorded in the third group (Figure 2).

Based on the results of the Kolmogorov-Smirnov test for normality distribution, it was found that all analyzed variables, except vWf activity, do not follow a normal distribution. Accordingly, the Kruskal-Wallis test was applied to determine the differences in mean values of HbA1c, fasting glucose, postprandial glucose, TAC, and GFR across the three participant groups. The results of the Kruskal-Wallis test and its corresponding p-value led to the conclusion that there was a statistically significant difference in the average values of HbA1c, postprandial glucose, and GFR among the three groups (p<0.05). However, there was no statistically significant difference in the mean value of TAC (p>0.05) (Table 1).

As previously mentioned, vWf activity was the only variable that followed a normal distribution. Therefore, a parametric F-test (ANOVA) was used to determine differences in its mean values among the three groups (Table 2).

**Table 2:** Results of Tukey's post hoc test ANOVA: analysis of variance; SD: standard deviation

Analyzed variables	Participants group	Number of participants	Mean ± SD	Min	Max	Variation range	ANOVA F-test	df	P-value
von Willebrand factor	1–5 years	46	1.08 ± 0.45	0.00	2.48	2.48	5.26	(2:132)	0.006
	6–10 years	49	1.20 ± 0.29	0.51	1.95	1.44			
	More than 10 years	40	1.36 ± 0.43	0.56	2.13	1.57			
	Total	175	1.17 ± 0.39	0.00	2.48	2.48			

The lowest mean value of the von Willebrand factor activity was recorded in the first group (1.08), while the highest was found in the third group (1.36). There was a statistically significant difference in the mean values of the vWf activity between the three groups (p<0.05). Tukey’s post hoc test indicated that a statistically significant difference exists only between the first and third groups at a 5% level of significance.

## Discussion

Our study results show that there was no statistically significant difference in fasting glucose values among the three groups (p=0.057), while postprandial glucose levels showed a statistically significant difference (p<0.001). Other studies have shown a greater variation in fasting glucose levels, which is associated with the onset of microalbuminuria and consequently lower GFR values [11,12]. Our study confirmed that GFR decreases with longer disease duration, with a statistically significant difference observed between the groups (p=0.037). This finding aligns with previous studies indicating that patients with T2DM for more than 10 years tend to have lower GFR values [13,14].

We did not find a statistically significant difference in TAC among the three groups (p=0.464), although other studies have suggested that lower TAC levels are associated with an increased risk of diabetes and its complications. TAC has been shown to correlate with antioxidant levels, and dietary recommendations for diabetes prevention and treatment should consider antioxidant intake [15]. Diabetes is a condition characterized by increased oxidative stress, which reduces total antioxidant capacity and necessitates higher antioxidant intake [16]. Our findings, however, did not confirm a statistically significant difference in TAC levels between the groups. The study by Pawar et al. [17] showed significantly lower TAC levels in patients with longer disease duration. HbA1c values increase with the duration of the disease, with the highest values observed in participants with a disease duration longer than 10 years. A statistically significant difference was found between the three groups (p<0.048). These findings are consistent with the results of other studies [18], which have indicated that diabetes-related complications are not solely associated with longer disease duration, but are also correlated with elevated levels of HbA1c and the presence of proteinuria.

The study by Nusca et al. reported that HbA1c can identify participants at higher risk of thrombosis, and it is well known that participants with T2DM are at increased risk for thromboembolic events [19-22]. Similarly, the study by Ma et al. [22] emphasized the association between elevated HbA1c levels and chronic diabetic complications. Other research has demonstrated that increased HbA1c values can serve as predictors of diabetes-related complications [23], and findings from another study [11] confirmed that HbA1c levels outside the target range are the strongest predictors of myocardial infarction in these participants. Our results also support these findings, as vWf activity levels were shown to increase with disease duration, with a statistically significant difference observed among the three groups (p<0.006). This suggests a greater risk of cardiovascular complications in participants with diabetes. The study by Peng et al. [23] found that vWf values are significantly higher in participants with T2DM who have developed complications compared to those without complications.

Our study demonstrated a statistically significant difference in HbA1c, postprandial glucose (two hours after meal), and GFR among the three participant groups (p<0.05). The study by Zhang et al. [24] showed a significant correlation between creatinine levels, diabetes duration, and HbA1c. Furthermore, results from other studies suggest that eGFR is associated with the presence and severity of diabetes-related complications [25]. Our findings confirm that there is a statistically significant relationship between the duration of diabetes and HbA1c, GFR, and vWf activity values.

## Conclusions

In our cohort, higher levels of vWf activity were found in people who had been treated for type 2 diabetes for more than 10 years. These results indicate that the level of vWf activity in people with type 2 diabetes 10 years after the onset of the disease can be used as a marker of vascular pathology. Therefore, routine measurement of vWf activity levels may aid in risk stratification and therapeutic decisions in individuals treated for type 2 diabetes for more than 10 years. Based on our findings, prospective studies with a larger number of subjects and surveys on dietary habits, physical activity, and the education level of subjects can be planned in order to better understand glycemic control and the prevention of diabetes complications.
